# Bone and Muscle Crosstalk in Aging

**DOI:** 10.3389/fcell.2020.585644

**Published:** 2020-12-10

**Authors:** Chen He, Wenzhen He, Jing Hou, Kaixuan Chen, Mei Huang, Mi Yang, Xianghang Luo, Changjun Li

**Affiliations:** ^1^Department of Endocrinology, Endocrinology Research Center, Xiangya Hospital of Central South University, Changsha, China; ^2^National Clinical Research Center for Geriatric Disorders, Xiangya Hospital of Central South University, Changsha, China; ^3^Key Laboratory of Organ Injury, Aging and Regenerative Medicine of Hunan Province, Changsha, China

**Keywords:** bone, muscle, crosstalk, aging, osteosarcopenia

## Abstract

Osteoporosis and sarcopenia are two age-related diseases that affect the quality of life in the elderly. Initially, they were thought to be two independent diseases; however, recently, increasing basic and clinical data suggest that skeletal muscle and bone are both spatially and metabolically connected. The term “osteosarcopenia” is used to define a condition of synergy of low bone mineral density with muscle atrophy and hypofunction. Bone and muscle cells secrete several factors, such as cytokines, myokines, and osteokines, into the circulation to influence the biological and pathological activities in local and distant organs and cells. Recent studies reveal that extracellular vesicles containing microRNAs derived from senescent skeletal muscle and bone cells can also be transported and aid in regulating bone-muscle crosstalk. In this review, we summarize the age-related changes in the secretome and extracellular vesicle-microRNAs secreted by the muscle and bone, and discuss their interactions between muscle and bone cells during aging.

## Introduction

With increasing aging populations, studies regarding aging have become an emerging field. Aging is the process of tissue deterioration coupled with an enormous economic burden. Osteosarcopenia is an age-related pathological condition characterized by porous and fragile bone and sarcopenia (a disease exhibiting low muscle mass and function), recognized as a “hazardous duet,” which induces fragile bones and increased risk of fractures, thus leading to high mortality rates and global financial threat. Binkley and Buehring (2009) first coined Sarco-Osteopenia to diagnose simultaneous bone and muscle weakness, calling for scientists to investigate specific mechanisms of the co-existence of osteoporosis and sarcopenia. Fat infiltration is a common character of aged bone and muscle; besides, osteoporosis and sarcopenia share several genetic, developmental, and endocrine factors. Endocrine factors have become a hot spot for studying the pathogenesis of osteosarcopenia, especially regarding myokines and osteokines.

Myokines, defined as soluble molecules expressed and released by muscle fibers, regulate the biological and pathological activities of local and distant cells and organs. McPherron et al. (1997) identified the first myokine, i.e., myostatin, which is expressed in developing and mature muscle and negatively regulates muscle mass. Steensberg et al. (2000) reported that contracting muscle releases interleukin-6 into the bloodstream, and realized that muscle could function as an endocrine organ. Moreover, with the development of modern sequencing and analytic technology, about 672 myokines were identified (Grube et al., 2018). They are composed mainly of peptides, such as growth factors, cytokines, and some small organic acids. Various myokines in the circulation work together to maintain homeostasis via muscle-organ crosstalk (Das et al., 2020). For example, contracting muscle secretes brain-derived neurotrophic factors to improve the memory and learning capacity, and exercise-induced irisin was reported to promote thermogenesis and increase bone mass (Vaynman et al., 2004; Boström et al., 2012; Colaianni et al., 2015; Severinsen and Pedersen, 2020).

Osteokines, a combination of two Greek words (“osteo” meaning “bone” and “kino” meaning “movement”), represent bone cell-derived factors, which influence local and systemic metabolism. The bone marrow microenvironment is complex and comprises diverse cells. Cells that participate in bone metabolism include bone marrow mesenchymal stem cells (BMSCs), osteoblasts, osteocytes, osteoclasts, and their precursor cells. The most widely studied osteokines are of osteoblast, osteocyte, and osteoclast origin. For instance, receptor activator of nuclear factor kappa B ligand (RANKL) and osteoprotegerin (OPG) from osteoblasts, sclerostin (SOST), fibroblast growth factor 23 (FGF23), and RANKL from osteocytes, and receptor activator of nuclear factor kappa B (RANK) from osteoclasts etc. Unlike skeletal muscle, from which over 600 myokines were identified, bone was not recognized as an endocrine organ until 2007 (Lee et al., 2007). In addition, the research regarding bone-derived factors mediating bone-muscle crosstalk is limited.

Besides the cytokines, growth factors and proteases released by aging cells, which are defined as the senescence-associated secretory phenotype (SASP), are harmful to musculoskeletal function. The nuclear acid changes in aging cells reportedly induce dysfunction in their parent cells (Yang et al., 2017; Li et al., 2018; Xiao Y.Z. et al., 2020). Recently, extracellular vesicles holding the above factors shed from senescent cells have also been recognized as another type of SASP (Alibhai et al., 2020).

Extracellular vesicles play an important role in cell-to-cell communication, as they can circulate in blood and dock into distant target cells to exert regulatory roles. Extracellular vesicles (EVs) can be divided into many types depending on their size, synthesis, and secretion mechanisms. Current methods cannot isolate profoundly pure subsets; therefore, we prefer the umbrella term “extracellular vesicles.” Two main subtypes of EVs exist, including exosomes, which are “cup shaped” membranous vesicles between 30 and 150 nm released by late endosome fusion with plasma membranes, and microvesicles, irregularly shaped vesicles measuring 100–1,000 nm, which originate directly from cell membranes. EVs hold microRNAs, proteins, mRNAs, lipids, etc., bind with or are endocytosed by recipient cells, transport their cargo to target cells to modulate biological activities. Recently, many articles describe the regulatory effects of senescent cell-released EVs in various pathologic conditions. Kadota et al. (2018) had reviewed the effects that senescent-cell-EVs exert on age-related lung diseases. In addition, enhanced miR-29b-3p expression in senescent-BMSC-EVs accounts for insulin resistance during aging (Su et al., 2019). However, there are few researches investigating the effects of EVs released from aging cells on musculoskeletal homeostasis. For instance, researchers found that raising miR-31 in aged BMSC and endothelial cell EVs suppress bone formation and enhance bone resorption, likewise increased miR-31 could inhibit dystrophin response to loading, and cause muscles of aged rats more susceptible to injury after disuse (Weilner et al., 2016; Hughes et al., 2018; Xu R. et al., 2018).

**FIGURE 1:**
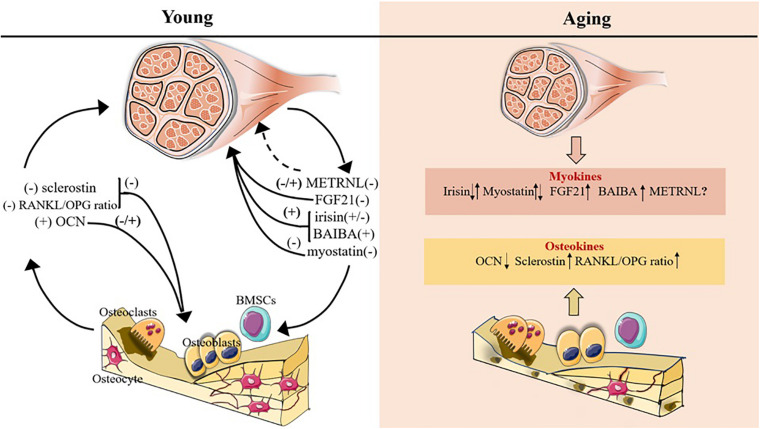
Myokines and osteokines altered with age in bone-muscle crosstalk. The plus symbol indicates positive association with bone/muscle mass; minus symbol indicates negative association with bone/muscle mass; upward and downward arrows indicate expression increase and decrease, respectively; question mark and the dotted line indicate that the function on bone/muscle is unclear.

**FIGURE 2:**
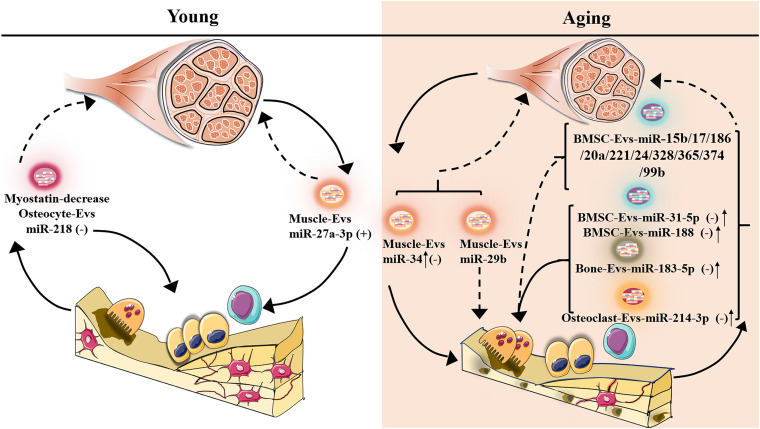
Potentially different expression of senescent-EV-microRNAs in bone-muscle crosstalk. The plus symbol indicates positive association with bone/muscle mass; minus symbol indicates negative association with bone/muscle mass; upward arrow indicates expression increase; the dotted line indicates that the function on bone/muscle is unclear.

There are many reviews regarding musculoskeletal communication for osteosarcopenia, but they mainly focus on simple bone-muscle crosstalk; aging and nutrition are included as additional factors to emphasize that dysfunction of skeletal muscle and bone always occur together (Reginster et al., 2016; Li et al., 2019). In this review, we focus on bone-muscle crosstalk during aging, briefly describe several classical and new myokines and osteokines (Tables 1, 2 and Figure 1), discuss how EV-microRNAs change with age (Table 3 and Figure 2), and finally point out the shortcomings of present studies and provide prospects of the potential field in age-related musculoskeletal diseases.

**TABLE 1:** Myokines altered with age in bone-muscle crosstalk.

	Target organ/cell and age-related bone-muscle crosstalk	Main physiology function
**Myokines**		
Irisin	BMSCs	Increase ALP positive colonies (Colaianni et al., 2014). Promote BMSCs develop into osteogenic lineage via ATF4-dependent RUNX2 activation (Colaianni et al., 2015). Rescue colony formation and ALP, Collagen I expression of BMSCs from suspended mice (Colaianni et al., 2017).
	Osteoblasts	Promote osteoblast proliferation and differentiation via MAPK pathway (Colaianni et al., 2015; Qiao et al., 2016). Reduce osteoblast apoptosis (Xu et al., 2020). Preserve osteoblast differentiation via β-Catenin in suspended mice (Chen et al., 2020). Polarize Macrophages into M2 phenotype via AMPK pathway then promote osteoblast differentiation (Ye et al., 2020).
	Osteocytes	Assist osteocyte-related osteocytic osteolysis and increase sclerostin expression (Kim et al., 2018). Downregulate SOST expression (Storlino et al., 2020). Inhibit the apoptosis of osteocytes under oxidative stress and/or microgravity via decreasing mitochondrial pathway of cell death (Storlino et al., 2020). Regulate osteoclast formation and activities via modulating OPG expression (Colaianni et al., 2017).
	Osteoclasts	Reduce expression of Cathepsin K, *Rank* and *Nfatc1* mRNA level (Ma et al., 2018). Promote proliferation and decrease differentiation of osteoclast precursors through p38, JNK and RANKL-induced NF-κB pathway separately (Ma et al., 2018).
	Muscle	Enhance the mRNA level of muscle growth-related gene like *Igf1* and *Pgc1*α*4* through ERK signaling pathway and suppress the expression of myostatin (Huh et al., 2014). Improve the proliferation and fusion of myoblasts; enhance protein synthesis via activation of AKT and ERK; activate satellite cells pool; increase expression of *Il-6*, then facilitate myogenesis (Reza et al., 2017b). Improve sarcolemmal stability or increase phosphorylation of FOXO3α and attenuate chymotrypsin-like enzyme activity (Reza et al., 2017a; Chang and Kong, 2020). Promote proliferation of C2C12 cells via increasing chemokine (C-C motif) ligand 7 (Lee et al., 2019).
	Age-related bone-muscle crosstalk	Negatively correlated with age (Huh et al., 2012; Rana et al., 2014). Increase with age (Mahmoodnia et al., 2016; Ruan et al., 2019). Use as a biomarker to predict pre-sarcopenia and sarcopenia (Chang et al., 2017). Positive association with bone mineral status and low irisin level is related to increased hip fracture risk (Yan et al., 2018).
Myostatin	BMSCs	Promote mesenchymal multipotent cells differentiate toward adipocytes (Hamrick et al., 2007). Induce the expression of *Ppar*γ and *C/EBP*α (Rebbapragada et al., 2003). Decrease the mechanosensitivity of BMSCs (Hamrick et al., 2007).
	Osteoblasts	Inhibit expression of *Alp*, osteocalcin, osterix and *Runx2* (Chen et al., 2017; Zhang et al., 2020). Decrease the number of osteoblasts on bone surface (Chen et al., 2017). Low-intensity pulsed ultrasound inhibits myostatin to enhance the proliferation of osteoblasts (Sun et al., 2019).
	Osteocytes	Increase the expression of Wnt pathway inhibitor SOST, DKK1, besides, promote RANKL expression in osteocytes (Qin et al., 2017). Reduce the miR-218 expression in osteocytes-derived exosomes and then impairs osteoblastic differentiation via Wnt pathway (Qin et al., 2017).
	Osteoclasts	Increase the expression of *Nfatc1* and the number of TRAP + multinucleated giant cells (Chen et al., 2017). Enhance expression of integrin αv, integrin β3 and calcitonin receptor via enhancing SMAD2-dependent NFATc1 nuclear translocation (Dankbar et al., 2015).
	Muscle	Myostatin-null mice show muscle hypertrophy (Shan et al., 2013). Myostatin inhibition by GASP-2 can promote proliferation and differentiation of myoblasts (Perie et al., 2016). Downregulate *Pax7* to suppress activation and self-renew of quiescent satellite cells; arrest C2C12 myoblasts in G1 phase through promoting P21 expression; decrease *Pax3*, *Myod1*, and *Myf5* expression to suppress differentiation (Rodriguez et al., 2014). Enhance ribosome biogenesis through activating S6K and rpS6 (Rodriguez et al., 2014). Decrease protein synthesis via inhibiting eEF2K-eEF2 after AMPK phosphonation (Deng et al., 2017). Promote the gene expression of *Murf-1*, enhance the proteasome activity of 26S and increase the expression of autophagy-related genes, such as *Atg3, Atg12*, etc. (Wang D.T. et al., 2015).
	Age-related bone-muscle crosstalk	Increase with age until 57 years old, then it exhibits a decrease trend (Szulc et al., 2012). Myostatin level is periodic, and exhibit the highest concentration in spring (Szulc et al., 2012). Increase or decrease with age (Bowser et al., 2013; Poggioli et al., 2016). Myostatin in fast muscle fiber ensued with aging (Shibaguchi et al., 2018). *Mstn* deficiency improve muscle atrophy and reduced muscle capacity in aging mice (Siriett et al., 2006). Myostatin inhibitor can be used to treat age-related sarcopenia (White and LeBrasseur, 2014). High myostatin level is related to low BMD subjects in Chinese elderly (Wu et al., 2018). Myostatin inhibition enhance osteogenesis induced by aged myofibers and myoblast cell line (Zhang et al., 2020).
FGF21	BMSCs	Favor BMSCs commitment into adipocytes over osteoblast lineage via PPARγ-FGF21 feed-forward regulatory pathway (Wei et al., 2012).
	Osteoclasts	Bone resorption enhanced in *Fgf21* transgenic mice possibly via increasing the expression level of RANKL in osteocytes (Wei et al., 2012). Promote RANKL-induced osteoclastogenesis via promoting IGFBP1 release by liver then combination with Integrin β1 receptor in osteoclasts (Wang X. et al., 2015). MiR-100 overexpression causes reduced FGF21 level thus inhibit osteoclasts-mediated bone resorption and partially restore the bone phenotype in OVX-operated mice (Zhou et al., 2019).
	Muscle	Dispensable for muscle mass maintenances in normal condition and necessary to induce muscle atrophy and weakness in starvation state via enhancing BNIP3 activation (Oost et al., 2019). Reduction of OPA1 is related to aging-related muscle atrophy, while *Fgf21* deletion is reported to partially rescue the muscle loss caused by OPA1 deficiency (Tezze et al., 2017). Induce cell cycle exit in C2C12 myoblasts through suppressing the cell cycle-related protein sequential to activation of p21 induced by p53 (Liu S. et al., 2017; Liu et al., 2017b. Enhance myogenesis via activating PAX3, and promote formation of smaller aerobic myofibers during fasting via the FGF21-SIRT1-AMPK-PGC1α pathway (Liu S. et al., 2017; Liu et al., 2017a,b)
	Age-related bone-muscle crosstalk	Increase with age (Hao et al., 2018; Lee et al., 2020). Negative associated with BMD (Hao et al., 2018; Lee et al., 2020). Positive relevant with the sarcopenia (Hanks et al., 2015; Tezze et al., 2017).
BAIBA	Osteoblasts	Promote proliferation or enhance the expression of *Runx2, Opg*, osteopontin, and *Alp* via moderate ROS induced by NAD(P)H oxidase 4 (Zhu X.W. et al., 2018).
	Osteocytes	Protect young osteocytes from ROS induced cell death via protecting the mitochondrial morphology and function (Kitase et al., 2018).
	Muscle	BAIBA can prevent loss of EDL and soleus muscle function in unloading hindlimb male mice (Jung et al., 2015). BAIBA prevent hyperlipidemia-induced insulin resistance and palmitate-induced inflammation in C2C12 myoblast cell line (Kitase et al., 2018).
	Age-related bone-muscle crosstalk	The protective effect of BAIBA on osteocytes decreasing with age due to reduction of BAIBA receptor-MRGPRD in osteocytes (Kitase et al., 2018). Aged skeletal muscle produces more BAIBA (Kitase et al., 2018).
METRNL	Osteoblasts	Inhibit mineralized nodule formation (Gong et al., 2016).
	Muscle	Wait to explore.
	Age-related bone-muscle crosstalk	Wait to explore.

**TABLE 2:** Osteokines altered with age in bone-muscle crosstalk.

	Target organ/cell and age-related bone-muscle crosstalk	Main physiology function
**Osteokines**		
Osteocalcin	Muscle	Improve the ability of muscle to utilize glucose and fatty acids and form a feed-forward axis to amplify the role in adaption to exercise (Mera et al., 2016a). Necessary to maintain muscle mass in old mice via promoting protein synthesis (Mera et al., 2016b). Enhance C2C12 myoblast cell proliferation and myogenic differentiation thorough activation of the PI3K/Akt, p38 and GPRC6A-ERK1/2 signaling pathway separately (Liu S. et al., 2017). Positive associated with muscle mass or function (Levinger et al., 2014; Harslof et al., 2016; Lin et al., 2016).
	Bone	Marker of bone formation, but *Ocn*^–/–^ mice exhibit an increased bone mass (Li J. et al., 2016). Suppress bone formation when coupled to a Gi-protein in human osteoblast cell line (Bodine and Komm, 1999). Promote bone mineral mature in an *Ocn* knockout mice and hydroxyapatite crystal growth was suppressed by OCN in certain studies (Neve et al., 2013). Recruit osteoclasts and promote osteoclastogenesis or enhance its chemotaxis (Neve et al., 2013; Li J. et al., 2016). Enhanced RANKL-dependent bone resorption mediated by muscle released IL-6 (Mera et al., 2016a).
	Age-related bone-muscle crosstalk	Decrease with age (Mera et al., 2016a; Diemar et al., 2020). Negative association with BMD (Kim et al., 2010; Ling et al., 2016). Maintain muscle mass and improve the muscle phenotype of aged mice (Mera et al., 2016a,b)
Sclerostin	Muscle	*Sost ^–/–^* mice exhibit an increasing trend of lean body mass fraction and overexpress sclerostin by an adeno-associated virus showing a significant reduction in lean body mass (Kim S.P. et al., 2017). Sclerostin is higher in low muscle mass subjects than healthy controls (Kim et al., 2019). Suppress Wnt3a mediated crosstalk between the MLO-Y4 osteocytes and muscle cells C2C12 via regulating Wnt/β-catenin pathway (Huang et al., 2017). Sclerostin inhibition restore muscle function in cancer induced muscle weakness (Hesse et al., 2019). Cannot prevent soleus muscle atrophy in rodents after spinal cord injury, probably because minimally expression of *Lrp5*/*Lrp6* in the rat soleus (Phillips et al., 2018).
	Bone	Various modifications in or near *SOST* can cause abnormal bone mineral density such as Sclerosteosis and Van Buchem disease (Holdsworth et al., 2019). Sclerostin transgenic mice exhibit osteopenia (Winkler et al., 2003). Regulate differentiation and mineralization of osteogenic lineage cell via a MEPE-ASARM dependent mechanism (Atkins et al., 2011). Sclerostin inhibition could reactive quiescent bone lining cells (Kim S.W. et al., 2017).
	Age-related bone-muscle crosstalk	Ensues with aging (Mirza et al., 2010; Ota et al., 2013). Lower sclerostin level in osteoporosis patients (Reppe et al., 2015). Ablation of *Sost* showed an increasing trend of lean body mass in elderly animals (Kim S.P. et al., 2017). A positive relevance between sclerostin and sarcopenia in type 2 diabetes mellitus (Medeiros et al., 2020).
OPG/RANKL/RANK	Muscle	RANKL-RANK interaction activates NF-κB, a pro-inflammation pathway which can inhibit myogenic differentiation and enhance the expression of ubiquitin proteasome system to induce muscle atrophy. Anti-RANKL protect muscle from chronic inflammation and improve its mechanical properties through shifting macrophages into M2 phenotype and inhibit the activation of NF-κB (Hamoudi et al., 2019). Dystrophic mdx mice specifically knockout *Rank* in muscle rescue its maximum specific force, while full-length OPG-Fc treatment activates sarco/endoplasmic reticulum Ca^2+^ ATPase to regulate Ca^2+^ homeostasis in dystrophic EDL through another pathway (Dufresne et al., 2018). RANKL inhibitor Dmab can rescue muscle function in human (Bonnet et al., 2019). *Opg* knockout mice displayed selective atrophy of fast-twitch-type IIb myofibers atrophy and C2C12 myotube exhibited reduced cross section area when exposure to RANKL (Hamoudi et al., 2020).
	Bone	RANKL-RANK activate major molecules in downstream pathway signaling, like nuclear factor NF-κB and colony stimulating factor 1 receptor et al., thereby driven pre-osteoclasts to differentiate into osteoclasts (Chung et al., 2014). *Opg* ablated mice showed increased adipocytes and BMSCs from these mice prefer to adipogenesis via enhanced expression of adipogenic transcription marker, *Ppar*γ (Zhang et al., 2016). RANK in osteoclasts derived exosomes induce reverse RANKL signaling leading to increased osteogenesis and mineralization (Ikebuchi et al., 2018).
	Age-related bone-muscle crosstalk	RANKL/OPG ratio, a preferable harmful determinant of bone mass, increase with age (Chung et al., 2014; Zhang et al., 2016; Piemontese et al., 2017). IL-3 can improve the RANKL/OPG ratio and act as a potent medicine to treat osteoporosis (Singh et al., 2018). RANKL inhibitor Dmab can rescue muscle function in postmenopausal women, and also in osteo-sarcopenic *Pparb*^–/–^ mice (Bonnet et al., 2019).

**TABLE 3:** Potentially different expression of senescence-EVs-microRNAs in bone-muscle crosstalk.

	Target organ/cell and age-related bone-muscle crosstalk	Main physiology function
EVs-microRNAs from skeletal muscle	Bone	MiR-27a-3p in muscle-EVs promote MC3T3-E1 osteogenic differentiation via β-catenin signaling pathway (Takafuji et al., 2020). Negative effect on osteoclast formation and its mitochondrial metabolism (Fulzele et al., 2019).
	Muscle	EVs-miR-27 might be an active player, and wait to explore.
	Age-related bone-muscle crosstalk	MiR-34a is highly express in aged mouse skeletal muscle and oxidative stress treated C2C12 cells, then these EVs home to bone marrow leading to BMSCs senescence via repressing SIRT1 expression (Yang et al., 2020). Role of EV-miR-1, miR-133a, miR-27, miR-29b-3p, and miR-34a in osteosarcopenia wait to explore.
EVs-microRNAs from bone cells	Bone	Osteocytes-derived EVs miR-218 can be suppressed by myostatin, then taken up by osteoblasts inhibiting osteoblastic differentiation (Qin et al., 2017).
	Muscle	Wait to explore.
	Age-related bone-muscle crosstalk	Aged BMSCs express high level of miR-31a-5p leading to bias lineage fate of BMSCs to adipocytes through SATB2, and it release exosomes with high level of miR-31a-5p, then increase the number and function of osteoclasts via Rho pathway (Xu R. et al., 2018). Aged osteoclasts were found to liberate EVs unloading enhanced expression of miR-214-3p, increasing miR-214-3p not only promote osteoclast formation but also embracing in osteoclasts-derived EVs then lead to impaired bone formation (Li D. et al., 2016). MiR-183-5p increase with age in bone-derived EVs and endocytosed by BMSCs to suppress osteogenic differentiation and lead to senescence (Davis et al., 2017). Role of miR-15b, miR-17, miR-183, miR-186, miR-188, miR-20a, miR-214, miR-221, miR-24, miR-31, miR-328, miR-365, and miR-374, and miR-99b etc. in aged bone cells-EVs in osteosarcopenia wait to explore.

## Proteome Altered With Age in Bone-Muscle Crosstalk

### Myokine

#### Irisin

Irisin, a polypeptide comprising 112 amino acids, is a messenger derived from skeletal muscle to regulate metabolism. Boström et al. (2012) revealed that irisin, a proteolytic product with amino terminal residues of fibronectin type III domain containing 5 (FNDC5), acts as an exercise-induced myokine to promote thermogenesis by browning white fat. Besides, Boström et al. (2012) revealed that irisin might function in glucolipid metabolism. Lately, it has been confirmed to rescue obesity in high-fat diet animal models and improve insulin resistance by targeting genes involved in glucose transport and utilization, such as solute carrier family 2, member 4 (*Glut4*) and phosphoenolpyruvate carboxykinase (*Pck*) (Xin et al., 2016). In addition, irisin was reported to promote pancreatic β cell proliferation and protect it from apoptosis to directly maintain pancreatic function. Exercise increases serum irisin level, furthermore, exercise is of great benefit to bone remodeling. Therefore, irisin was chosen for further investigation to determine whether it is engaged in muscle-bone communication *in vitro* and *in vivo*.

##### The effects of irisin on BMSCs

Colaianni et al. (2014) reported that irisin is continuously released at a basal level by muscle and enhanced during exercise. BMSCs cultured in myoblast conditioned medium exhibit more alkaline phosphatase (ALP)-positive colonies (Colaianni et al., 2014). Furthermore, they found that recombinant irisin injection in young male mice once a week at a low dose of 100 μg/kg for 4 weeks increases cortical bone mass and stimulates osteoblastic bone formation without altering energy metabolism, indicating that irisin promotes expression of activating transcription factor 4 (*Atf4*), which subsequently enhances the biological activity of runt-related transcription factor 2 (RUNX2) and a series of osteogenic transcription factors, enabling BMSCs to develop into the osteogenic lineage (Colaianni et al., 2015). In addition, irisin can improve the colony formation ability of BMSCs and enhance the expression of previously impaired genes, such as *Alp* and collagen I, in hind-limb suspended mice (Colaianni et al., 2017).

##### The effects of irisin on osteoblasts

Increased bone formation not only can be realized by BMSC osteogenic bias but also promoted by enhanced osteoblast proliferation and differentiation. Mitogen-activated protein kinase (MAPK) signaling pathways mainly regulate cell proliferation and differentiation, with no exception regarding its role in osteoblasts. Qiao et al. (2016) illustrated that after treatment with irisin, basal MAPK14 (p38) and total MAPK1 (ERK) remain constant in osteoblasts, but expression levels of phosphorylated p38 and phosphorylated ERK increase and peak 5-20 min after stimulus. Pretreatment of primary rat osteoblasts and MC3T3-E1 cells with p38 inhibitor SB203580, or ERK inhibitor U0126 eliminate the effect of irisin on proliferation and differentiation (Qiao et al., 2016). Consistent with that, Colaianni et al. (2015) reported that irisin enhances ERK phosphorylation in cultured osteoblasts. Furthermore, irisin can reduce the number of apoptotic osteoblasts in an osteoporosis rat model, and protect against decreased osteoblast differentiation by increasing β-catenin levels (Chen et al., 2020; Xu et al., 2020). Except for its direct effect on osteoblasts, irisin can indirectly promote osteogenesis by polarizing macrophages to the M2 phenotype via the adenosine monophosphate-activated protein kinase (AMPK) pathway (Ye et al., 2020).

##### The effects of irisin on osteocytes

Kim et al. (2018) first discovered the irisin receptor, αV Integrin, in osteocytes. They reported that an osteocyte-related direct bone resorption model, ovariectomy (OVX)-induced Osteocytic osteolysis, requires *Fndc5* and sclerostin to induce bone resorption. Moreover, sclerostin, a protein specifically expressed in osteocytes, appears increased in a dose-dependent manner after culture with irisin (Kim et al., 2018). Nevertheless, Colaianni et al. (2015) showed that irisin level is negatively correlated with sclerostin. Kim et al. (2018) hypothesized that irisin plays a bidirectional role depending on its exposure mode, like the parathyroid hormone (PTH). Lately, Storlino et al. (2020) demonstrated that SOST expression is downregulated by intermittent irisin administration, while continuous irisin exposure doesn’t modify SOST expression. Moreover, they found that irisin inhibits the apoptosis of osteocytes under oxidative stress and/or microgravity (Storlino et al., 2020). Apoptosis inhibition can be explained by the decreased mitochondrial (intrinsic) pathway of cell death. Specifically, irisin can enhance the expression of mitochondrial transcription factor A and increase the ratio of B cell leukemia 2 (BCL2)/BCL2-associated X protein (BAX), while high BCL2 level can impair BAX initiating autoactivation of caspase-9, then block the activation of the executioner, caspase-3. Besides, it can improve the expression of Podoplanin and Connexin 43, genes important for osteocyte dendrite and gap junction channel formation, respectively (Storlino et al., 2020). In addition, irisin may target osteocytes first, then modulates OPG expression, thus decreasing the RANKL/OPG ratio to regulate osteoclast formation and activities (Colaianni et al., 2017).

##### The effects of irisin on osteoclasts

Besides the effects on BMSCs and osteoblasts etc., Colaianni et al. (2015) also found that irisin-treated mice exhibit significantly lower number of osteoclasts. Consistent with the above result, irisin was found to reduce the phosphatase acid tartrate resistant (TRAP)-positive multinucleated cells induced by disuse, and it can reduce expression of Cathepsin K, *Rank*, and nuclear factor of activated T cells cytoplasmic 1 (*Nfatc1*) mRNA levels significantly (Ma et al., 2018). The detailed mechanism may be explained by the fact that irisin promotes proliferation and decreases differentiation of osteoclast precursors through the p38, c-Jun amino terminal kinase (JNK) signaling pathways and the RANKL-induced nuclear factor kappa B (NF-κB) pathway (Ma et al., 2018). Furthermore, Ibanez et al. (2014) and Xu et al. (2020) found that irisin could upregulate nuclear factor E2-related factor 2 (NRF2) expression levels, while *Nrf2* knockout increases the survival rate of osteoclasts. This indicates that irisin might decrease the survival of osteoclasts via *Nrf2* modification.

##### The effects of irisin on muscle

Besides paracrine and endocrine functions, irisin has also been shown to promote myogenesis via autocrine function. Huh et al. (2014) demonstrated that irisin expression increases during myogenic differentiation, furthermore, irisin exposure enhances mRNA levels of muscle growth-related genes, such as insulin-like growth factor 1 (*Igf1*) and peroxisome proliferative activated receptor gamma coactivator 1 alpha4 (*Pgc1*α*4*) through the ERK signaling pathway, and suppresses myostatin expression (Huh et al., 2014). An *in vivo* study conducted by Reza et al. (2017b) showed that irisin injection in normal and pathogenic circumstances cause significant skeletal muscle hypertrophy, accompanied with enhanced grip strength. The underlying mechanisms include (1) irisin improves proliferation and fusion activity of myoblasts; (2) enhances protein synthesis by promoting activation of AKT and ERK; (3) expands the activated satellite cells pool; (4) increases expression of exercise-related genes, such as interleukin-6, thereafter facilitating myogenesis (Reza et al., 2017b). In addition, irisin can partially restore muscle atrophy caused by various etiologies by improving sarcolemmal stability or increasing phosphorylation of forkhead box O3 alpha (FOXO3α) and attenuating chymotrypsin-like enzyme activity (Reza et al., 2017a; Chang and Kong, 2020). Moreover, irisin administration can enhance C2C12 myoblasts expressing FNDC5, suggesting that irisin might amplify its positive effect on myogenesis through a regeneration feedback loop (Colaianni et al., 2015). Furthermore, irisin promote C2C12 proliferation via increased chemokine (C-C motif) ligand 7 of active downstream ERK pathway (Lee et al., 2019). However, further investigation is required regarding irisin receptors in muscle cells to understand the mechanisms behind the activation of different signaling pathways.

##### Irisin in bone-muscle crosstalk during aging

During aging, decreased mobility and loss of muscle may reduce irisin expression. This hypothesis laid a foundation for follow-up studies investigating the relationship between irisin and aging together with age-related musculoskeletal diseases. The relationship between irisin and age is controversial, with irrelevant, positive, and negative correlation. Huh et al. (2012) reported that irisin level is negatively correlated with age, and it may be induced by age-related muscle loss. Consistent with these results, multiple regression analysis models using backward elimination showed that telomere length is inversely proportional to age and positively associated with plasma irisin level. Telomere length can be predicted by plasma irisin level, and this suggested that age may be negatively associated with irisin level (Rana et al., 2014). Likewise, many epidemiological studies refer to circulating irisin levels growing with age (Mahmoodnia et al., 2016; Ruan et al., 2019). The paradoxical results may be due to researchers using different ELISA kits, which produce varying results, or the inclusion and exclusion criteria set by the researchers differ. More attention should be paid to confirm the genuine relationship between these parameters. In addition, irisin level is positively correlated to human biceps circumference, and irisin was confirmed to use as a biomarker to predict pre-sarcopenia and sarcopenia (Huh et al., 2012; Chang et al., 2017). Furthermore, irisin level is positively associated with bone mineral status, while low irisin level is related to increased hip fracture risk, further corroborating that age-related decreases in muscle and bone may be explained by decreased irisin level, but the detailed mechanism of action remains to be elucidated (Yan et al., 2018).

#### Myostatin

Myostatin, also known as growth differentiation factor 8 (GDF8), is a transforming growth factor-beta (TGFβ) superfamily protein mainly secreted from muscle fibers. Myostatin is released in an inactive form, then undergoes a series of proteolytic cleavage by bone morphogenetic protein 1/tolloid family of metalloproteinases, and finally produces a mature polypeptide. The hydrolysates N-terminal prodomain can interact with the mature homodimer, counteracting myostatin binding to activin type IIB receptor and activin-like kinase 4 (ALK4) or activin-like kinase 5 (ALK5) heterodimer. Furthermore, follistatin can also combine with myostatin and competitively inhibit myostatin binding to the receptor (Rebbapragada et al., 2003; White and LeBrasseur, 2014). Myostatin is a negative regulator of muscle and strength, and genetic mutation or ablation of the myostatin (*Mstn*) leads to muscle hypertrophy, enhanced strength, and improved bone architecture (Dankbar et al., 2015). The elaborate biological functions of myostatin in the musculoskeletal system are described below.

##### The effects of myostatin on BMSCs

The “colony switch” refers to the fact that once bone marrow stromal cells favor osteogenic differentiation, adipogenesis will be adversely affected. Hamrick et al. (2007) illustrated that BMSCs cultured within an osteogenic medium (obtained from *Mstn* knockout mice) exhibit more pronounced ALP and alizarin red staining than WT controls, and myostatin exposure induces mesenchymal multipotent cells differentiation toward adipocytes. Consistent with that, Rebbapragada et al. (2003) demonstrated that myostatin induces the expression of adipogenic transcription factors peroxisome proliferator-activated receptor gamma (*Ppar*γ) and CCAAT enhancer binding protein alpha (*C/EBP*α). Although myostatin ablation promotes osteogenic bias, it cannot restore unloading-induced bone catabolism, which may be due to myostatin-reduced osteogenic differentiation via decreased mechanosensitivity of BMSCs (Hamrick et al., 2007).

##### The effects of myostatin on osteoblasts

Unsurprisingly, *in vitro* exogenous GDF8 can inhibit the expression of *Alp*, osteocalcin, and two important osteogenic transcription factors, osterix and *Runx2*, while alizarin red staining is also restrained. Moreover, *in vivo*, recombinant myostatin decreases the number of osteoblasts on the bone surface, while its neutralizing antibody reverses this effect (Chen et al., 2017). Myostatin inhibition can rescue the ALP signal and RUNX2 expression in aged primary myofiber- and C2C12 cell line-treated MC3T3-E1 (Zhang et al., 2020). This occurs because the myostatin inhibitor combines with myostatin to reduce negative effects from aged muscle on osteogenic differentiation. Recently, Sun et al. (2019) find that low-intensity pulsed ultrasound could improve bone healing by regulating the myostatin signaling pathway, they deduced that low-intensity pulsed ultrasound promote osteoblast proliferation via inhibiting Smad3-induced β-catenin stabilization through compromised myostatin signaling pathway.

##### The effects of myostatin on osteocytes

Research has reported that myostatin directly affects osteocytes. Osteocytes, comprising 90% of total bone cells, are deeply embedded in the bone matrix, thus the research on primary osteocytes is limited. Qin et al. (2017) used Osteocytic (Ocy454) cells as an osteocyte-study model, and revealed that myostatin could increase the expression of Wnt pathway inhibitor SOST, dickkopf Wnt signaling pathway inhibitor 1 (DKK1), while promoting the osteoclastogenesis gene *Rankl* in osteocytes. Moreover, myostatin can reduce miR-218 expression in osteocyte-derived EVs and its parent cells, while miR-218 was reported to promote the Wnt pathway by inhibiting SOST expression. Specifically, EVs released by myostatin-treated Ocy454 cells can be taken up by osteoblasts and osteoclast precursor cells during cultured within a medium, and then it can impair osteoblastic differentiation via the Wnt pathway, but exert no effect on osteoclastic differentiation (Qin et al., 2017). This conclusion was made based on *in vitro* results, and *in vivo* study is required further.

##### The effects of myostatin on osteoclasts

Besides affecting the expression of RANKL in osteocytes, myostatin can also directly increase the expression of osteoclast differentiation-related genes. Myostatin incubation with RANKL and macrophage colony stimulating factor (M-CSF) treated macrophages increases the number of TRAP^+^ multinucleated giant cells, while it also increases the expression of NFATc1 in a dose-dependent manner (Chen et al., 2017). Likewise, bone surface osteoclasts appeared reduced in myostatin antibody-administrated mice (Chen et al., 2017). Recently, Dankbar et al. (2015) demonstrate that *Mstn* knockout mice display lower osteoclast number and smaller osteoclast size, but it seems like myostatin exerts no effect on osteoclast function *per se*, they also report that myostatin enhances the expression of genes engaged in RANKL-induced osteoclastogenesis, such as Integrin αv, Integrin β3, and calcitonin receptor, via enhancing SMAD2-dependent NFATc1 nuclear translocation, which is independent from the ERK pathway.

##### The effects of myostatin on muscle

Shan et al. (2013) revealed that myostatin-null mice show muscle hypertrophy. Inhibiting the myostatin canonical pathway by growth and differentiation factor-associated serum protein 2 (GASP-2) was shown to promote proliferation and differentiation of myoblasts (Perie et al., 2016). Regulation of myostatin in myogenesis is profoundly complicated. Rodriguez et al. (2014) elaborated on the mechanism via two schematic illustrations. First, myostatin downregulates paired box 7 (*Pax7*) to suppress activation and self-renewal of quiescent satellite cells. Second, C2C12 myoblasts are arrested in the G1 phase through promotion of p21 expression and inhibition of phosphorylated cyclin dependent kinases 2 (CDK2) and cyclin dependent kinases 4 (CDK4). Third, myostatin suppresses differentiation by reducing myogenic genes, such as *Pax3*, myogenic differentiation 1 (*Myod1*), and myogenic factor 5 (*Myf5*), through downregulation of the MEK/ERK1/2 MAPK pathway and/or the AKT/mTORC1 signaling pathway (Rodriguez et al., 2014). Besides affecting the number of muscle fibers, myostatin also decreases its cytoplasmic volume by disrupting the balance between protein synthesis and degradation. Specifically, besides sequential activation of transcription factors eukaryotic translation initiation factor 4E (eIF4E) and eukaryotic translation initiation factor 4E binding protein 1 (4E-BP1) in the PI3K/Akt/mTOR pathway, myostatin inhibition was found to affect translation efficiency and ability by enhancing ribosome biogenesis via the activation of ribosomal protein S6 kinase (S6K) and ribosomal protein S6 (rpS6) (Rodriguez et al., 2014). In addition, myostatin can decrease protein synthesis through inhibition of eukaryotic translation elongation factor 2 kinase (eEF2K)-eukaryotic translation elongation factor 2 (Eef2) after AMPK phosphonation (Deng et al., 2017). As for protein degradation, it was reported that myostatin not only promotes gene expression in ubiquitin proteasome, such as tripartite motif-containing 63 (*Murf-1*), and enhances the proteasome activity of 26S; but also increases the expression of autophagy-related 3 (*Atg3*), *Atg12*, etc., which are autophagy-related genes (Wang D.T. et al., 2015).

##### Myostatin in bone-muscle crosstalk during aging

Shibaguchi et al. (2018) showed that myostatin increases with age in fast muscle fibers, while myostatin and its receptors in slow muscle are less responsive. This may explain why fast muscle fiber atrophy is characteristic of aging muscle. Opposing results may be explained by species differences (Bowser et al., 2013). Moreover, muscle atrophy and reduced muscle capacity are improved in aging *Mstn-*deficient mice compared to WT littermates (Siriett et al., 2006). Further, evidence indicates that the myostatin inhibitor can treat age-related sarcopenia (White and LeBrasseur, 2014). Nevertheless, the relevance between age and myostatin is ambiguous. Different opinions exist, with some reporting that circulating myostatin level increases with age until 57 years old, and decreases thereafter, while others implied that serum myostatin increases or decreases with age continuously (Szulc et al., 2012; Bowser et al., 2013; Poggioli et al., 2016). Interestingly, Szulc et al. (2012) found that myostatin level exhibits circannual variation, with greater concentrations observed during spring. These various opinions may due to a highly homologous amino acid sequence-growth and differentiation factor 11 (GDF11), to date no effective method can distinguish GDF11 from GDF8, furthermore, many studies examined circulating total myostatin, including the inactive and active forms, but only biologically active forms are functional. Circulating myostatin levels do not reflect the actual amount in special tissues (Szulc et al., 2012; Shibaguchi et al., 2018; Wu et al., 2018). Moreover, western blot analysis may not reflect true levels of myostatin, as other proteins may appear at the same molecular weight (Poggioli et al., 2016). Regarding the relationship between altered myostatin level and bone status, a cross-sectional study illustrated that high mature myostatin level is significantly related to low bone mineral density (BMD) in elderly Chinese subjects (Wu et al., 2018). Meanwhile, aged myofibers and myoblast cell line enhance osteogenesis upon exposure to myostatin propeptides, which indicates that myostatin may be a potential target to treat osteosarcopenia. Initially, the safety of the myostatin inhibitor was controversial, but later Morissette et al. (2009) dispelled fears that using myostatin inhibitor could cause heart enlargement and showed that it improves insulin sensitivity and bone density (Zhang et al., 2020). In addition, Ran et al. (2020) revealed that anchoring the propeptide to the second extracellular loop of the exosome surface marker CD63 could assist the impaired administration efficacy due to low biological activity and stability. Further research is required to promote myostatin inhibitor as a promising prospect for patients with osteosarcopenia.

#### Other Myokines

Other myokines, such as fibroblast growth factor 21 (FGF21), β-aminoisobutyric acid (BAIBA), and Meteorin-like (METRNL), have been discovered in recent years and reported to affect osteogenesis, myogenesis, and bone-muscle crosstalk during aging. Here, we briefly describe their roles in bone and muscle, and their effects on age-related crosstalk.

##### Fibroblast growth factor 21

Fibroblast growth factor 21, abbreviated as FGF21, is weakly expresses in healthy muscle, and it increases with age, starvation, and mitochondrial dysfunction (Oost et al., 2019). FGF21 belongs to the FGF family, scientists have found 23 FGF proteins to date, most FGF family proteins regulate cell proliferation, while FGF21 mainly affects metabolism (Itoh, 2014). Despite expressed in muscle, it is also referred to as a hepatokine and adipokine, as the primary organ releasing FGF21 depends on different stimuli (Itoh, 2014). The function of FGF21 on metabolism is controversial, as scientists found that *Fgf21* knockout prevents high-fat ketogenic diet-induced insulin resistance in mice, but others confirmed the benefits of FGF21 on glucolipid metabolism (Itoh, 2014). Also, the relationship between FGF21 and bone and muscle is intriguing.

###### The effects of FGF21 on osteogenesis

Wei et al. (2012) firstly identified FGF21 as a skeletal metabolism regulator in 2012, they reported that either FGF21 pharmacological exposure or genetic gain-of-function, causes significantly low bone mass phenotype, moreover, PPARγ-FGF21 forms a feed-forward regulatory pathway, which favors BMSCs commitment to adipocytes instead of osteoblast lineage. In addition, they found that osteoclast-mediated bone resorption increases in *Fgf21* transgenic mice and suggested that FGF21 may promote osteoclastogenesis by increasing RANKL expression level in osteocytes (Wei et al., 2012). Charoenphandhu et al. (2017) and Hao et al. (2018) sequentially confirmed the deterioration effect of FGF21 on skeletal and the indirect influence of FGF21 on osteoclasts in humans and rats. Wang X. et al. (2015) discovered that FGF21 mediates a liver-osteoclast communication via insulin-like growth factor binding protein 1 (IGFBP1). FGF21 enhances the expression of IGFBP1 mainly in the liver, and then IGFBP1 is released into the bloodstream, promotes RANKL-induced osteoclastogenesis via combination with Integrin β1 receptor, further activates RANKL-induced NFATc1 expression (Wang X. et al., 2015). Furthermore, miR-100 was identified as the upstream regulator of *Fgf21*, miR-100 overexpression reduces FGF21 levels in serum and liver, thus inhibiting osteoclast-mediated bone resorption and partially restoring the bone phenotype in OVX-operated mice (Zhou et al., 2019). Nevertheless, none of these studies investigated the direct effects of muscle-derived FGF21 on bone and bone-muscle crosstalk.

###### The effects of FGF21 on myogenesis

Recently, attention is turned to the autocrine function of myokine FGF21. Evidence showed that FGF21 requires other factors to trigger its harmful function in muscle. Oost et al. (2019) demonstrated that *Fgf21* is dispensable for muscle mass maintenance under normal conditions, while it is necessary to induce muscle atrophy and weakness in a starvation state via enhancing mitophagic flux mediated by activation of mitophagy protein BCL2/adenovirus E1B interacting protein 3 (BNIP3). Reduction of the important pro-fusion protein optic atrophy protein 1 (OPA1) is related to aging-related muscle atrophy, Tezze et al. (2017) found that in double muscle-specific *Opa1/Fgf21* knockout mice, *Fgf21* deletion could partially rescue the muscle loss caused by OPA1 deficiency. These observations suggested a harmful effect of FGF21 on muscle. Nevertheless, FGF21 administration cannot inhibit the FGF19 function, which induces muscle hypertrophy (Benoit et al., 2017). Furthermore, FGF21 was found to induce cell cycle exit in C2C12 myoblasts by suppressing the cell cycle-related protein by p21, which is activated by p53 (Liu S. et al., 2017; Liu et al., 2017b). In addition, Liu et al. (2017a) suggested that FGF21 enhances myogenesis through PAX3 activation, and it could promote formation of aerobic myofibers via the FGF21-sirtuin-AMPK-peroxisome proliferative activated receptor, gamma coactivator 1 alpha (PGC1α) pathway *in vitro* and *in vivo*. The effect of FGF21 on myogenesis is obscure, and scientists should focus on deciphering these effects to discover novel therapeutic intervention strategies aimed at treating musculoskeletal diseases.

###### FGF21 in bone-muscle crosstalk during aging

FGF21 was found to increase with age, and reported to be negatively associated with BMD (Hao et al., 2018; Lee et al., 2020). The mechanism behind this may be attributed to BMSCs preferring to form adipocytes over osteoblasts or osteoclasts enhancing its bone resorption via FGF21-IGFBP1 axis (Wei et al., 2012; Wang X. et al., 2015); however, Lee et al. (2020) rejected the second half of the hypothesis and demonstrated that age-related bone loss caused by FGF21 is irrelevant to the FGF21-IGFBP1 axis. Further, serum FGF21 was reportedly positively associated with sarcopenia (Hanks et al., 2015; Tezze et al., 2017). The major shortcoming of this research regarding the relationship between FGF21 with BMD and muscle mass is the cross-sectional study design, as it cannot conclude a causal relationship between age-related changes in FGF21 and bone or muscle loss in the elderly. Therefore, further research is required to investigate whether FGF21 can be used as a potential target to treat osteosarcopenia, and the causal correlation should be explored.

##### β-aminoisobutyric acid

BAIBA refers to β-aminoisobutyric acid, which has two enantiomers, L-BAIBA and D-BAIBA, like every other amino acid except for glycine. BAIBA was found in urine and reported as a myokine generated during exercise or from muscle-PGC-1α-over-expressing mice. It is a catalysate of branched chain amino acid valine and thymine, exhibiting effects on browning white adipose tissue and promoting β-oxidation of fatty acid in liver (Roberts et al., 2014). Reports have shown that BAIBA improves metabolism dependent on PPAR and leptin (Kammoun and Febbraio, 2014; Roberts et al., 2014). As a newly discovered exercise-induced myokine, it has attracted many scientists to study its mysterious role in bone-muscle crosstalk.

###### The effects of BAIBA on osteogenesis

Kitase et al. (2018) found that BAIBA, especially L-BAIBA, could protect young osteocytes from reactive oxygen species (ROS)-induced cell death by protecting mitochondrial morphology and function, and reduce bone loss in a hind-limb unloading model. Subsequently, Zhu X.W. et al. (2018) demonstrated that appropriate low doses of BAIBA either promotes proliferation or enhances the expression of osteogenic transcription factor RUNX2 and differentiation markers OPG, osteopontin, and ALP in MC3T3-E1 osteoprogenitor cells. Meanwhile, they declared that moderate ROS production by NAD(P)H oxidase 4 might explain the BAIBA stimulatory effect on an osteoblast cell line (Zhu X.W. et al., 2018). Recently, Wang et al. (2020) illustrate that BAIBA is positively associated with BMD in young, healthy women. The correlation is disrupted after the occurrence of a fracture or during an obese state, mainly due to interference caused by medical treatments and cytokines released by obese subjects. Research regarding the effects of BAIBA on bone is far from enough, whether it can affect formation or function of osteoclasts remains to be studied, and its physiological dosages for bone formation also need to be determined.

###### The effects of BAIBA on myogenesis

Very few BAIBA functional studies on muscle exist. Scientists found that BAIBA could prevent loss of extensor digitorum longus (EDL) and soleus muscle function in unloading hind-limb male mice (Kitase et al., 2018). While BAIBA increases the male EDL muscle mass only, it indicated that gains in muscle mass are unnecessary for improved muscle function and gender specific hormone expression of female mice might account for unaffected muscle phenotype (Kitase et al., 2018). Furthermore, the only *in vitro* study on the effects of BAIBA on muscle reported that BAIBA prevents hyperlipidemia-induced insulin resistance and palmitate-induced inflammation in a C2C12 myoblast cell line (Jung et al., 2015). Although it was widely recognized that insulin resistance and muscle inflammation are two mainly harmful factors to muscle mass and function, further research is required to investigate the direct effect of BAIBA on myogenesis (Cleasby et al., 2016).

###### BAIBA in bone-muscle crosstalk during aging

Reactive oxygen species-induced osteocyte cell death could be rescued by BAIBA treatment. To our knowledge, increased ROS can lead to aging, therefore BAIBA, which protects cells from ROS injury, may be a profoundly effective osteoporosis treatment (Hamrick and McGee-Lawrence, 2018). The protective effect of BAIBA on osteocytes decreases with age; to further elucidate the underlying mechanism, researchers compared not only the ability of skeletal muscle to produce BAIBA in different-aged mice but also the expression of BAIBA receptor-Mas-related G-protein-coupled receptor type D (MRGPRD) in young and aged osteocytes (Kitase et al., 2018). Interestingly, Kitase et al. (2018) demonstrated that decreasing level of MRGPRD in osteocyte membranes could account for the compromised effect of BAIBA on aged osteocytes, although BAIBA production increases in aged skeletal muscle. Further investigations should be conducted to explore methods to improve MRGPRD expression in osteocytes. Further, the detailed role of BAIBA in osteosarcopenia deserves exploration.

##### Meteorin-like

Meteorin-like is a small protein molecule reported to regulate glucose metabolism, thermogenesis, and inflammation (Ushach et al., 2018). Scientists using *Pgc-1*α splice isoform *Pgc-1*α*4* muscle-specific transgenic mice reported that METRNL is released by muscle after exercise; in addition, they found adipocytes can also release METRNL during cold stimulation (Rao et al., 2014). METRNL recruits eosinophils to secret IL-4/IL-13, activating macrophages to release anti-inflammatory cytokines to improve glucose homeostasis and promote white adipose tissue (WAT) browning (Rao et al., 2014). In addition, METRNL, also defined as Subfatin, appears downregulated in adipose tissue of calorie restricted rats and is highly expressed in obese-mice adipocytes and during adipogenic differentiation, thus thought to be an adipokine related to energy storage (Li et al., 2014). Moreover, METRNL was found to be an anti-inflammatory cytokine expressed in human barrier tissue and macrophages, while *Metrnl* knockout mice do not exhibit abnormal metabolism but exhibit immunity defects (Ushach et al., 2018). The reason for this phenomenon may be that, like FGF21, METRNL is weakly expressed under normal conditions and mainly plays roles in regulating immunity, it requires other factors to trigger its function regarding energy metabolism. Furthermore, research investigating the roles of METRNL in bone and muscle regulation is not well-developed, with only one study reporting that METRNL could affect Ap-1 transcription factor and inhibit mineralized nodule formation (Gong et al., 2016). No studies exist regarding the effects of METRNL on skeletal muscle mass and function, but research has shown that METRNL recruits eosinophils to activate macrophages, and eosinophil-macrophage synergy reportedly participates in repairing damaged muscle (Heredia et al., 2013; Rao et al., 2014). Therefore, METRNL supposedly affects muscle regeneration and/or myogenesis. This indicates that METRNL may function as a messenger to mediate muscle-bone crosstalk and their interplay during aging.

### Osteokines

#### Osteocalcin

Osteocalcin (OCN), a secreted protein originating from osteoblasts, goes through sequential cleavages to split amino acid sequences, such as signal peptides, and obtains γ-carboxylate at three residues aided by a cofactor – vitamin K. There are two forms of OCN depending on the degree of carboxylation, while the under-carboxylated form is considered to function in blood circulation, which stems from an acid circumstance caused by bone matrix resorption through osteoclasts or directly released by osteoblasts during acute stress response (Battafarano et al., 2020). OCN is like hormones in biological characteristic and physiological function. Serum OCN can enter distant cells to regulate insulin resistance, glucose homeostasis, male fertility, and brain function (Li J. et al., 2016; Mizokami et al., 2017; Battafarano et al., 2020). Recently, many studies show that OCN acts as a messenger and participates in interactions between bone and muscle. OCN expression increases dramatically during bone matrix mineralization, which is considered to be a marker representing late osteogenesis. Besides, skeletal muscle, a locomotive and glycogen storage organ, is also an important target tissue of OCN.

##### The effects of osteocalcin on muscle

Initially, studies investigating OCN effects on skeletal muscle were limited to energy metabolism. Mera et al. (2016a) demonstrated that OCN could improve muscle to utilize glucose and fatty acids, meanwhile, they found that mice deficient in *Ocn* exhibit impaired exercise capacity and exogenous OCN administration could rescue the exercise capacity of aged mice. Furthermore, OCN could stimulate skeletal muscle to release IL-6, simultaneously IL-6 and OCN form a feed-forward axis to amplify adaption to exercise (Mera et al., 2016a). Next, the influence of OCN on muscle mass was investigated. Using female mice in a study, to exclude the effect of testosterone, Mera et al. (2016b) found that osteocalcin signaling is necessary to maintain muscle mass in old mice by promoting protein synthesis. However, this research did not elaborate on the mechanism. Later, Liu S. et al. (2017) illustrated and confirmed that under-carboxylated OCN enhances C2C12 myoblast cell proliferation and myogenic differentiation thorough activation of the PI3K/Akt, p38, and GPRC6A-ERK1/2 signaling pathways, respectively. Moreover, several investigations on rats, healthy volunteers, or patients with hypoparathyroidism revealed that under-carboxylated OCN is positively associated with muscle mass or function (Levinger et al., 2014; Harslof et al., 2016; Lin et al., 2016). However, this experiment should be repeated in a large healthy population.

##### The effects of osteocalcin on bone

The effects of OCN on bone are divided into three parts. First, the controversial role in bone formation. OCN is highly expressed in mature osteoblasts and a familiar marker of bone formation, however, unexpectedly, *Ocn*^–/–^ mice exhibit increased bone mass (Li J. et al., 2016). Bodine and Komm (1999) found an OCN receptor coupled with a Gi-protein in a human osteoblast cell line and confirmed that OCN could suppress bone formation. However, the specific mechanism has not been clarified. Second, the role in bone mineralization. OCN has a strong affinity for Ca^2+^ and hydroxyapatite and reportedly promotes bone mineral maturity in *Ocn* knockout mice; furthermore, hydroxyapatite crystal growth is suppressed by OCN in certain studies (Neve et al., 2013). Third, the role in bone resorption, besides recruiting osteoclasts and enhancing its chemotaxis, OCN fragments can also promote osteoclastogenesis (Neve et al., 2013; Li J. et al., 2016). Mera et al. (2016a) further demonstrated that OCN could stimulate skeletal muscle release IL-6, subsequently increasing the expression of RANKL in osteoblasts and decreasing the decoy receptor of *Rankl*, in other words, OCN enhances bone resorption mediated by IL-6 released from muscle. The detailed mechanism should be elucidated, and researchers should distinguish the functional component between the fragments or carboxylation status.

##### Osteocalcin in bone-muscle crosstalk during aging

It is widely recognized that sex hormones and brain function decline with age. Studies reported positive association between serum OCN and testosterone, meanwhile low OCN is related to impaired brain cognition and microstructure (Puig et al., 2016; Zhong et al., 2016). Therefore, although the results regarding OCN and age are controversial, we prefer the theory suggesting plasma OCN levels decrease with age (Mera et al., 2016a; Diemar et al., 2020). Nevertheless, many cross-sectional investigations revealed that OCN is negatively associated with BMD (Kim et al., 2010; Ling et al., 2016). The effects of OCN may be blurred by diverse subtypes, which may explain the contradictory results between age-related bone loss and age-related decreases in OCN, and its deleterious function on bone. Thus, further study is required to confirm whether OCN can be used as a treatment for osteoporosis and sarcopenia.

#### Sclerostin

Sclerostin is a glycoprotein released by osteocytes and recognized as a member of Dan/Cerberus family of bone morphogenetic protein (BMP) antagonists. Sclerostin primarily originates from mature osteocytes, but is also observed in the cardio-cerebral vascular system, lung, and kidney, with only the mRNA expression detected in the latter tissues (Delgado-Calle et al., 2017; Holdsworth et al., 2019). Sclerostin not only couples with BMP to suppress BMP-induced phosphorylation of Smad but also impairs the canonical Wnt pathway through competitive combination with LDL receptor related protein 5/6 (LRP5/6), the co-receptor of the Wnt pathway (Winkler et al., 2003; Fijalkowski et al., 2016). In addition, the binding of sclerostin to LRP5/6 requires the “molecular chaperone” LRP4; consequently, mutations in sclerostin or *Lrp4* produce similar abnormal phenotype (Fijalkowski et al., 2016).

##### The effects of sclerostin on muscle

Sclerostin may play an important role in muscle mass modulation. von Maltzahn et al. (2012) reported that the Wnt signaling pathway is engaged in the differentiation of muscle stem cells. Moreover, Kim S.P. et al. (2017) showed that *Sost*^–/–^ mice exhibit increased lean body mass in older animals with *P-*value of 0.06, while over-expressing sclerostin by an adeno-associated virus revealed the opposite body composition phenotype in *Sost*^–/–^ mice, which showed an increase in body fat mass and a small, but significant reduction in lean body mass. Further, circulating sclerostin levels are higher in low muscle mass subjects than healthy controls (Kim et al., 2019). In addition, Huang et al. (2017) found that sclerostin could suppress WNT3a mediated crosstalk between MLO-Y4 osteocytes and muscle cells (C2C12) by regulating the Wnt/β-catenin pathway. While a sclerostin inhibitor enhances muscle regeneration, it can also restore muscle function in cancer induced muscle weakness, but it cannot prevent soleus muscle atrophy in rodents after spinal cord injury, possibly because low expression of *Lrp5/Lrp6* was observed in the rat soleus (Phillips et al., 2018; Hesse et al., 2019). A detailed mechanism of action regarding muscle function regulation by sclerostin should be explored further.

##### The effects of sclerostin on bone

*SOST* genetic mutation-induced high bone mass was reported in the 1950s, but the pathogenesis of the skeletal disease, named Sclerosteosis, was identified in 2001 (Van Buchem et al., 1955). Moreover, other modifications in or near *SOST* can cause abnormal BMD, such as Van Buchem disease (Holdsworth et al., 2019). In addition, sclerostin transgenic mice exhibit osteopenia (Winkler et al., 2003). Sclerostin is the second emerging therapeutic target, besides PTH, to promote bone anabolism. Several osteogenic activities, such as mechanical load or intermittent PTH administration, can influence serum sclerostin levels (Keller and Kneissel, 2005; Robling et al., 2008). Besides impairing the canonical Wnt pathway and BMP signaling pathway in bone formation, sclerostin was reported to regulate differentiation and mineralization of osteogenic lineage cells via a matrix extracellular phosphoglycoprotein (MEPE)-acidic serine aspartate-rich MEPE-associated motif (ASARM)-dependent mechanism (Atkins et al., 2011). Moreover, labeling osteoblasts with tamoxifen-inducible lineage-tracing strategies, researchers found that sclerostin inhibition could re-activate quiescent bone lining cells, but the underlying mechanism requires further investigation (Kim S.W. et al., 2017). Sclerostin not only affects the activities of osteogenic lineage cells within the bone microenvironment but also promotes bone resorption in a RANKL-dependent pathway in an autocrine manner (Carvalho et al., 2011).

##### Sclerostin in bone-muscle crosstalk during aging

Serum sclerostin levels ensue with aging, and osteoclasts are also a source of aging-related sclerostin increase (Mirza et al., 2010). It has been reported that osteoclast cultures from old mice produce more sclerostin than from young mice, suggesting that sclerostin may contribute to decreased bone formation in the elderly (Ota et al., 2013). However, Reppe et al. (2015) demonstrated that osteoporosis patients exhibit lower sclerostin levels than healthy controls. The relationship between sclerostin and bone mass is intriguing, since sclerostin inhibits bone formation and is produced by bone cells. There is a functional negative feedback between sclerostin and formation of mature osteocytes. Furthermore, Chang et al. (2014) found that LRP4 acts as an anchor to trap sclerostin in the local bone environment. Genetic deletion of *Lrp4* increases bone mass despite rising serum sclerostin, which indicated that circulating sclerostin level cannot reflect local bone turnover (Chang et al., 2014). Despite the ambiguous relationship between sclerostin level and aged-related osteoporosis, sclerostin inhibitor was found to increase bone mass and strength in the early stages of treatment in a pre-clinical study, although the effects were significantly reduced after prolonged treatment. Meanwhile, ablation of *Sost* increases lean body mass in elderly animals (Kim S.P. et al., 2017). In addition, it has been reported that diabetes is associated with increased risk of sarcopenia. Medeiros et al. (2020) reported on a study involving hemodialysis patients with diabetes and illustrated that sclerostin proportion is higher in patients with type 2 diabetes mellitus compared to healthy controls, possibly indicating that a positive relationship between sclerostin and sarcopenia exists. Thus, sclerostin may play an active role in age-related bone-muscle crosstalk.

#### OPG/RANKL/RANK

Receptor Activator of Nuclear Factor κB Ligand, abbreviated as RANKL, is a transmembrane protein located in osteocytes and osteoblasts. When talking about RANKL, it is necessary to introduce the OPG/RANKL/RANK system together. It is well known that bone formation and bone resorption are cyclical, dynamically balanced processes, but the molecular mechanisms that link bone formation and resorption were not substantially improved until 1997 (Simonet et al., 1997; Khosla, 2001). The identification of the OPG/RANKL/RANK system presented a new era in the history of bone biology. RANK is a tumor necrosis factor (TNF) receptor superfamily protein in osteoclasts, it can bind to RANKL to activate major molecules in downstream signaling pathways, such as nuclear factor kappa B (NF-κB) and colony-stimulating factor 1 receptor, thus driving differentiation of pre-osteoclasts to osteoclasts (Chung et al., 2014). Besides RANK, OPG also binds to RANKL, unlike other receptor proteins, it is a soluble receptor which suppresses osteoclast activation. Dysfunction of the OPG/RANKL/RANK axis can cause osteoporosis (Dufresne et al., 2018). In addition, RANK is reportedly expressed in skeletal muscle, while dysfunction of the OPG/RANKL/RANK axis induces various muscle phenotype, therefore, the OPG/RANKL/RANK axis presents a new avenue for the investigation of bone-muscle interplay (Dufresne et al., 2018).

##### The effects of OPG/RANKL/RANK on muscle

RANKL-RANK interaction can activate NF-κB, a pro-inflammation pathway, which can inhibit myogenic differentiation and enhance the expression of the ubiquitin proteasome system to induce muscle atrophy. Anti-RANKL treatment can shift macrophages into the M2 phenotype and inhibit the activation of NF-κB in Duchenne muscular dystrophy, thus protecting muscle from chronic inflammation and improving its mechanical properties (Hamoudi et al., 2019). In dystrophic mdx mice, *Rank* knockout rescues muscle strength, but is less effective than full-length OPG-Fc treatment. This may because while full-length OPG-Fc suppresses the RANK-RANKL combination, it also activates sarco/endoplasmic reticulum Ca^2+^ ATPase to regulate Ca^2+^ homeostasis in dystrophic EDL through another pathway (Dufresne et al., 2018). Moreover, Bonnet et al. (2019) observed that RANKL inhibitor Dmab could rescue muscle function in humans. Recently, Hamoudi et al. (2020) reveal that *Opg* knockout mice displays selective atrophy of fast-twitch-type IIb myofibers, while C2C12 myotubes exhibit reduced cross section area when exposed to RANKL, they further confirmed that the OPG/RANKL/RANK axis is involved in muscle function regulation.

##### The effects of OPG/RANKL/RANK on bone

The study of the OPG/RANKL/RANK system on bone began with the identification of OPG. Simonet et al. (1997) found that OPG is a TNF receptor molecule in the rat intestine, and over-expressing OPG in mice show a predominant decrease in osteoclasts (Khosla, 2001). Hormone deficiency and long-term use of glucocorticoids induced abnormal bone resorption are due to changes in RANKL or OPG expression (Singh et al., 2018). Traditionally, the OPG/RANKL/RANK axis reportedly plays a role in osteoclastogenesis. However, scientists found that OPG/RANKL/RANK has an entirely new role in regulating bone metabolism. Zhang et al. (2016) illustrated that *Opg* ablated mice show increased adipocytes, while BMSCs from these mice prefer adipogenesis via enhanced expression of adipogenic transcription marker, Pparγ. Recently, RANK is found to play a role in osteoclast-to-osteoblast crosstalk via EVs released from osteoclasts to induce reverse RANKL signaling, leading to increased osteogenesis and mineralization (Ikebuchi et al., 2018).

##### OPG/RANKL/RANK in bone-muscle crosstalk during aging

The imbalance between bone formation and bone resorption leads to age-related osteoporosis (Yang et al., 2019). Much attention has been paid to the role of the OPG/RANKL/RANK axis in this process. Researchers found that RANK, RANKL, and RANKL/OPG ratio increase in the bone marrow with age, while the bone protective factor OPG decreases with age (Chung et al., 2014; Zhang et al., 2016). However, Piemontese et al. (2017) found that OPG increase with age in femoral marrow plasma, but it’s not as obvious as RANKL, indicating that the RANKL/OPG ratio would increase with age. Furthermore, Singh et al. (2018) demonstrated high serum RANKL/OPG ratio in adult mice, and IL-3 could improve the RANKL/OPG ratio and act as a potent medicine to treat osteoporosis. Therefore, changes in the RANKL/OPG ratio are more appropriate as a determinant of bone mass than individual changes in protein levels, such as RANKL or OPG alone. As for muscle, the relationship between RANKL/OPG/RANK and muscle atrophy has been reported, while the relationship with sarcopenia was rarely reported. Bonnet et al. (2019) observed that RANKL inhibitor Dmab could rescue muscle function in postmenopausal women, as well as in osteo-sarcopenic *Pparb*^–/–^ mice. This suggests that the OPG/RANKL/RANK axis may be an existing and feasible method to treat osteoporosis and sarcopenia together.

## Skeletal Muscle-Derived EV-microRNAs

### EV-microRNAs of Skeletal Muscle Engaged in Bone-Muscle Crosstalk

Skeletal muscle, a force generator in motion, also an essential endocrine organ, continually releases myokines and EVs to regulate organic metabolism and homeostasis.

A considerable amount of EVs circulate in the bloodstream, and muscle-derived EVs make up 1–5% of total EVs in circulation (Guescini et al., 2015). Guescini et al. (2015) illustrated that muscle-specific microRNAs enclosed in EVs are released into the blood during exercise, and 60–65% of the muscular EVs are CD81 positive. During moderate exercise, contracting muscle can strengthen bone quality and prolong lifespan. However, few studies reported that muscular EVs act on bone. Xu Q. et al. (2018) reported that EVs from C2C12 myoblasts or myotubes promote MC3T3-E1 cell osteogenic differentiation via the β-catenin signaling pathway, and miR-27a-3p is the major functional factor. Furthermore, miR-27a was found to enhance myoblast differentiation (Huang et al., 2012; Chen et al., 2014) and miR-27a mimic could rescue chronic kidney disease-induced muscle atrophy (Wang et al., 2017). This indicates that miR-27a in muscle-derived EVs might function in bone and muscle crosstalk. In addition, besides bone formation, muscular EVs also exert a negative effect on osteoclast formation and its mitochondrial metabolism, the latter is necessary for mature osteoclast resorption (Takafuji et al., 2020).

### EV-microRNAs of Skeletal Muscle in Bone-Muscle Crosstalk During Aging

Intensity and frequency of exercise are reduced in most elderly people, and senescent cell-derived EVs alter their cargo, contributing to age-related decline in somatic function. Alpha-sarcoglycan, an integral membrane glycoprotein, which specially localized to the sarcolemma acts as a typical marker of muscular EVs (Ervasti et al., 1990; Guescini et al., 2015). Recently, an intriguing study demonstrates that miR-34a is highly expressed in aged mouse skeletal muscle and serum EVs. Furthermore, Fulzele et al. (2019) reported that alpha-sarcoglycan-positive EVs and EVs from oxidative stress treated C2C12 cells all exhibit that miR-34a increases with age, and these EVs could home to bone marrow, leading to BMSC senescence by repressing SIRT1 expression. Elevated miR-34a in the aging muscle can account for muscle metabolic dysfunction, while decreased SIRT1 activity in aging muscles is associated with impaired muscle performance (Mohamed et al., 2014; Kukreti and Amuthavalli, 2020). Yang et al. (2020) reported that miR-29b-3p is increased in the plasma of aging mice and humans, differentiated atrophic C2C12 myotube could release miR-29b-3p-loaded EVs to induce neuronal dysfunction. miR-29b was shown to contribute to several types of muscle atrophy (Li et al., 2017). Moreover, regarding bone metabolism, miR-29b-3p was found to decrease in osteocytes under mechanical loading. Transfection of miR-29b-3p in osteocytes reduces IGF-1 production, thus suppressing osteoblast differentiation (Zeng et al., 2019). Future research should investigate whether miR-29b-3p in muscle-derived EVs acts as a critical factor in bone-muscle crosstalk during aging. Research should look into whether muscle-specific microRNAs enclosed in muscular EVs, which are influenced by exercise, such as miR-1, miR-133a, can be used for osteosarcopenia treatment (Guescini et al., 2015).

## Ev-microRNAs of Bone Cells Engaged in Bone-Muscle Crosstalk

Most scientists focused on the influence of muscle on bone mass, and sometimes on the effects of osteokines on muscle, while no studies directly investigated the influence of EVs secreted by bone on muscle. Herein, we summarize several microRNAs, which appear altered in senescent bone cell-derived EVs, reported to influence bone metabolism and regulate muscle metabolism.

A subset of differentially expressed EV-microRNAs among young and aging BMSCs were found recently (Davis et al., 2017; Umezu et al., 2017; Su et al., 2019). We summarize and select several from 65 age-dependent altered microRNAs, including miR-10a/10b-5p/15b/17-5p/182-5p/183-5p/185/186/196a-5p/20a/221/24/28-5p/31a-5p/328/365/374/411/467e-5p/487a/99b etc. By consulting articles regarding bone metabolism and myogenesis, we screen out four downregulated and seven upregulated microRNAs in aging BMSC-derived exosomes.

The downregulated microRNAs include miR-24, miR-328, miR-365, and miR-374. The commonality between these microRNAs downregulated in aging BMSC-EVs is that they reportedly benefit myogenesis and bone formation, or at least myogenesis. Sun et al. (2008) demonstrated that ectopic expression of miR-24 promotes the expression of myogenic differentiation markers in C2C12 myoblasts. Furthermore, miR-365 and miR-374 were reported to accelerate cardiomyocyte hypertrophy (Lee et al., 2017; Wu et al., 2017). Besides myogenesis, bone formation is also regulated by these two microRNAs. Concretely, miR-365 overexpression could improve the expression of *Runx2* in MC3T3-E1 cells and rescue dexamethasone-induced osteogenesis suppression; it can also suppress bone resorption via decreasing osteoclast number (Franceschetti et al., 2014; Xu et al., 2017). Regarding miR-374, Ge et al. (2018) found that miR-374 expression is significantly increased in fractured mice and verified that miR-374 improves MSC osteogenesis via PTEN using miR-374 transgenic and knockout model. Moreover, miR-328 may play an important role in bone-muscle crosstalk in aging. Liu D. et al. (2018) verified that apoptosis-deficient *MRL/lpr* and *Casp3*^–/–^ mice show impaired osteogenesis capability of BMSCs. Further, they found that circulating apoptotic bodies treatment maintains bone homeostasis of *MRL/lpr* and *Casp3*^–/–^ mice and improves the osteopenia phenotype of ovariectomized mice. Interestingly, Liu D. et al. (2018) found that miR-328 is a functional factor in apoptotic bodies. Lately, miR-328 was reportedly decreased in the plasma of the sarcopenia group in the elderly (Wu et al., 2017).

The upregulated microRNAs include miR-15b, miR-17, miR-20a, miR-186, miR-221, miR-31a-5p, and miR-99b. miR-15b was reported to be a negative regulator of myoblast differentiation via SET-domain containing 3 (SETD3) (Zhao et al., 2019). In addition, Fang et al. (2019) found that miR-15b could repress BMSC osteogenesis, while others revealed contrasting result that miR-15b has a positive effect on osteogenic differentiation, therefore, further research is required to determine if miR-15b is a potential target. miR-17 and miR-20a belong to the miR-17-92 cluster, which is reported to suppress myogenesis by reducing the expression of enigma homolog 1 (*Enh1*), then inhibiting myogenic regulatory factor (MRF) and E protein binding to the myogenesis genes (Qiu et al., 2016; Vimalraj et al., 2014). Moreover, Fang et al. (2016) found that silencing miR-17 could improve the micro-architecture of trabecular bones. Meanwhile, miR-17 and miR-20a were reported to enhance BMSC adipogenesis (An et al., 2016; Zhu E. et al., 2018). Nevertheless, Zhou et al. (2014) had shown that miR-17-92 cluster heterozygous mice exhibit worse trabecular micro-architecture than wild-type controls. Consequently, scientists should investigate whether miR-17 and miR-20a can act as a cluster enclosed in EVs to influence bone and muscle phenotype together in aging. Combined with the negative effect of miR-186 on rat BMSC osteogenesis and the inhibitory action on the final myogenesis differentiation step by influencing myogenin translation, we deduce that miR-186 may be the driving factor in bone-muscle crosstalk during aging (Antoniou et al., 2014; Xiao J. et al., 2020). Furthermore, the increased miR-221 in aged-BMSC may also function in osteosarcopenia, because it was reported that ectopic overexpression of miR-221 in skeletal muscle satellite cells inhibits myotube formation and miR-221 was found to be a negative regulator of bone formation (Zhang et al., 2017; Liu B. et al., 2018; Gan et al., 2020). Xu R. et al. (2018) reported aged BMSCs express high levels of miR-31a-5p leading to bias lineage fate of BMSCs to adipocytes through AT-rich sequence binding protein 2 (SATB2), aged BMSCs also release miR-31a-5p enriched EVs to its microenvironment, inducing increase in osteoclast number and function via the RhoA pathway. Moreover, age-dependent increases in miR-31 can inhibit the dystrophin response to loading, and cause muscles of aged rats more susceptible to injury after disuse (Hughes et al., 2018). Whether these above molecules can participate in age-related bone-muscle crosstalk or not requires further research. Furthermore, we were inspired to investigate whether miR-188, which appears increased in aging BMSCs, can be released through EVs to regulate aging muscle phenotype, since miR-188 was reportedly elevated in a muscular dystrophy dog model (Li et al., 2015; Shibasaki et al., 2019). Zacharewicz et al. (2014, 2020) reported that miR-99b displays a negative age-dependent association with protein synthesis in skeletal muscle. Next, they verified that overexpression of miR-99b in human primary myotubes could inhibit protein synthesis via regulatory-associated protein of mTOR (RPTOR). As for bone, Franceschetti et al. (2014) performed a microRNA array during osteoclastogenesis and found that miR-99b inhibition decreases both osteoclast number and size. Considering the effects of miR-99b on muscle and osteoclasts, we hypothesize that miR-99b might become a new target of osteosarcopenia in the elderly.

Besides BMSCs, aged osteoclasts were found to liberate EVs unloading enhanced expression of miR-214-3p comparing with the young. Increasing miR-214-3p not only directly promotes osteoclast formation but it’s also enclosed in osteoclast-derived EVs, then leading to impaired bone formation (Li D. et al., 2016). However, miR-214 was found to positively regulate C2C12 myoblasts proliferation and differentiation and reported to be an important participant in muscle metabolism (Feng et al., 2011). Overall, whether miR-214 can serve as a reasonable target to treat age-related osteoporosis and sarcopenia or not needs further exploration. In addition, Davis et al. (2017) collected bone marrow interstitial fluid from mice at two age stages and found that miR-183-5p increases with age in bone-derived EVs and is endocytosed by BMSCs, and finally suppresses osteogenic differentiation and induce BMSCs senescence. However, research is required on its function in muscle metabolism.

## Conclusion and Future Directions

There is much literature on bone and muscle crosstalk although, many key issues need to be addressed. Previous studies mainly focused on simple unidirectional communications between bone and muscle, little has reported their age-related reciprocal interactions. We outline age-related changes in the secretome and EV-microRNAs of bone and muscle, and aim to find out their common pathogenic factors and shared therapeutic targets. However, the study of age-related bone-muscle crosstalk is relatively complex, many uncharted fields still need to be explored.

Extracellular vesicles unloading many potential bioactive molecules act as information-transmitters between cells. Besides microRNAs, long non-coding RNA (LncRNA) is also enriched in EVs. No studies have reported its role in bone-muscle crosstalk in aging, thus researches on age-related LncRNA changes in BMSCs and muscle, together with their related LncRNA functional studies will provide a niche for further investigation (Nie et al., 2015; Li et al., 2018). Scientists can investigate whether aged bone cells can enclose LncRNA, such as Bmncr, within EVs to regulate musculoskeletal metabolism. Such studies will help to find efficient therapeutic applications in age-related musculoskeletal diseases.

## Author Contributions

CH wrote the manuscript and designed the figures. WH, JH, KC, MH, MY, and XL revised the manuscript. CL provided critical feedback and helped to shape the manuscript. All authors listed have made a substantial contribution to the work.

## Conflict of Interest

The authors declare that the research was conducted in the absence of any commercial or financial relationships that could be construed as a potential conflict of interest.
